# Patch-clamp analysis of nicotinic synapses whose strength straddles the firing threshold of rat sympathetic neurons

**DOI:** 10.3389/fnins.2022.869753

**Published:** 2022-10-04

**Authors:** Paul H. M. Kullmann, John P. Horn

**Affiliations:** Department of Neurobiology, School of Medicine, University of Pittsburgh, Pittsburgh, PA, United States

**Keywords:** sympathetic ganglia, EPSP, EPSC, synaptic integration, synaptic gain

## Abstract

Neurons in paravertebral sympathetic ganglia are innervated by converging nicotinic synapses of varying strength. Based upon intracellular recordings of excitatory postsynaptic potentials (EPSPs) with sharp microelectrodes these synapses were classified in the past as either primary (strong) or secondary (weak) by their ability to trigger postsynaptic action potentials. Here we present an analysis of 22 synapses whose strength straddled threshold, thereby distinguishing them from the original classification scheme for primary and secondary synapses. Recordings at 36°C were made from intact superior cervical ganglia isolated from 13 male and 3 female Sprague-Dawley rats and from 4 male spontaneously hypertensive (SHR) rats. Ganglia were pretreated with collagenase to permit patch recording. By dissecting a 1 cm length of the presynaptic cervical sympathetic nerve as part of the preparation and through use of graded presynaptic stimulation it was possible to fractionate synaptic inputs by their distinct latencies and magnitudes, and by the presynaptic stimulus threshold for each component. Comparison of cell-attached extracellular recordings with intracellular recordings of synaptic potentials and synaptic currents indicated that straddling EPSPs are not an artifact of shunting damage caused by intracellular recording. The results also showed that in cells where a single presynaptic shock elicits multiple action potentials, the response is driven by multiple synapses and not by repetitive postsynaptic firing. The conductance of straddling synapses also provides a direct estimate of the threshold synaptic conductance (9.8 nS ± 7.6 nS, *n* = 22, mean ± SD). The results are discussed in terms of their implications for ganglionic integration and an existing model of synaptic amplification.

## Introduction

The strengths of individual nicotinic synapses that converge on sympathetic neurons shape ganglionic integration. Intracellular recordings from isolated paravertebral sympathetic ganglia using sharp microelectrodes provided the original evidence for a dichotomy in which individual inputs to ganglionic neurons produced either very large nicotinic excitatory postsynaptic potentials (EPSPs) that invariably triggered action potentials or small nicotinic EPSPs that were subthreshold in strength (Dodd and Horn, 1983; Skok and Ivanov, 1983; Hirst and McLachlan, 1984; McLachlan, 2003). Strong suprathreshold EPSPs and weak subthreshold EPSPs were also evident in intracellular microelectrode recordings of natural activity from living animals (Skok and Ivanov, 1983; Ivanov and Skok, 1992; Ivanoff and Smith, 1995; McLachlan et al., 1997, 1998; Bratton et al., 2010). An analysis of B-type secretomotor sympathetic neurons in bullfrog sympathetic ganglia led to the naming of strong synapses as primary and of weak synapses as secondary (Karila and Horn, 2000). These authors then proposed an *n* + 1 rule based on the bullfrog data and on the literature describing mammalian paravertebral ganglia. The rule posited that sympathetic neurons in paravertebral ganglia receive one primary synapse and a variable number, *n*, of secondary synapses. Models incorporating *n* + 1 convergence make the interesting prediction that ganglionic integration can produce use-dependent amplification of preganglionic activity (Karila and Horn, 2000; Wheeler et al., 2004; Kullmann and Horn, 2006, 2010a; Kullmann et al., 2016).

Possible difficulties with existing models of synaptic amplification are raised by exceptions to the *n* + 1 rule found in mammalian sympathetic ganglia. In some neurons fluctuations in EPSP amplitudes during repetitive stimulation can straddle threshold and single presynaptic shocks sometimes elicit double spikes [cf. Figure 5 in Rimmer and Horn (2010)]. It remains unclear whether straddling synapses are physiological in origin or reflect damage caused by microelectrode impalement. Recording damage may also explain why several authors have interpreted microelectrode recordings as evidence that secondary EPSPs are too small to exert significant effect on ganglionic integration (Maslov et al., 1996; McLachlan, 2003; Janig, 2006). Evidence showing the consequences of a damage artifact comes from dynamic clamp experiments on dissociated rat sympathetic neurons using patch electrodes (Springer et al., 2015). Introducing a virtual non-depolarizing shunt conductance comparable to the damage associated with microelectrode impalement acts to lower the strength of virtual nicotinic EPSPs and can alter postsynaptic repetitive firing dynamics. Nonetheless, it remains unclear whether double spiking reflects repetitive firing or the impact of two or more converging synapses. To circumvent problems introduced by sharp microelectrodes, the present experiments exploit a technical advance that permits patch electrode recording from the isolated intact rat superior cervical ganglion (SCG). After establishing a giga-Ohm seal, one can record extracellularly without impalement damage and then intracellularly from the same neuron in the current-clamp and voltage-clamp modes. This approach permits the unambiguous identification of straddling synapses, and of synapses that can drive double spiking. It also provides the first direct estimate of the synaptic conductance required to reach the postsynaptic action potential threshold.

## Materials and methods

### Animals

All animal procedures were approved by the Institutional Animal Care and Use Committee (IACUC) at the University of Pittsburgh. Male and female Sprague Dawley (CD) rats and male SHR rats, aged 7–23 weeks, were obtained from Charles River (Wilmington, MA, United States). After killing rats by CO_2_ inhalation, superior cervical ganglia were rapidly dissected along with the adjacent carotid artery and transferred to a 60 mm Petri dish coated with Sylgard 184 (Dow Corning, Midland, MI, United States) and filled with Ringer solution for further preparation.

### Preparation of ganglia

**FIGURE 1:**
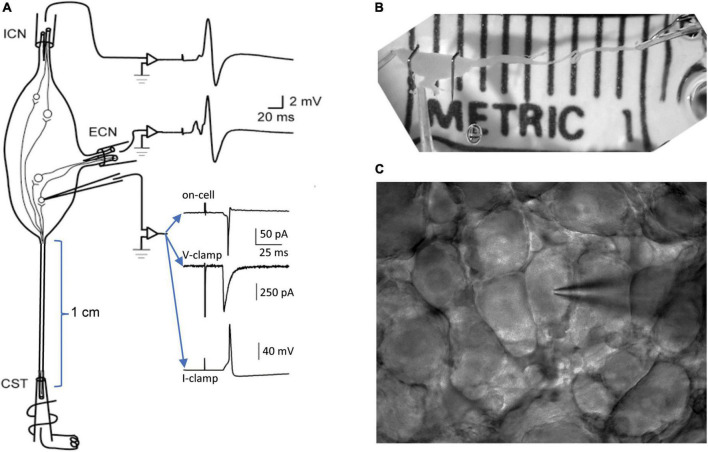
Methods for patch recording from the isolated superior cervical ganglion (SCG). **(A)** Schematic organization of the experimental preparation. Tight-fitting extracellular suction electrodes are applied to the preganglionic cervical sympathetic trunk (CST) and to the postganglionic internal carotid nerve (ICN) and external carotid nerve (ECN). Graded presynaptic stimulation is used to recruit and resolve converging synapses. The efficacy of presynaptic stimulation is monitored by recording compound extracellular responses from the ICN and ECN. A patch electrode is used to record extracellular and intracellular responses from individual neurons. Examples of recordings show an on-cell extracellular response, an intracellular synaptic current and an intracellular EPSP that triggers an action potential. **(B)** Photograph of a dissected rat SCG in the recording chamber after removal of the connective tissue sheath and application of suction electrodes. Note the long CST, which allows synaptic components to be separated by variations in axonal conduction velocities. Note also the two stainless pins gently holding down the ganglion. At this stage of preparation the ganglion is ready for collagenase treatment. **(C)** A field of sympathetic neurons and a patch electrode visualized through a 40× water immersion objective and charge-coupled device (CCD) camera, ready for recording.

This study extends previous methods for recording from the isolated intact SCG with microelectrodes (Li and Horn, 2006; Rimmer and Horn, 2010) and with patch electrodes (Springer et al., 2015). The SCG is situated in the neck at the rostral end of the paravertebral sympathetic chain, adjacent to the bifurcation of the carotid artery into its external and internal branches. By virtue of this anatomy (Figure 1A), presynaptic axons enter the ganglion through the cervical sympathetic trunk (CST) and postsynaptic axons exit through the external carotid nerve (ECN) and the internal carotid nerve (ICN). This fortuitous separation of inputs and outputs facilitates presynaptic stimulation and postsynaptic recording through separate extracellular suction electrodes (Figures 1A,B). When dissecting the ganglion, special care is taken to maximize the length of the associated nerves. In the rat, one can routinely dissect preparations where the CST is 1 cm or longer (Figure 1B) and the ECN and ICN are several mm long. To avoid injury during initial stages of the dissection, the preparation is handled by the attached carotid artery and connective tissue sheath. Maximizing the presynaptic conduction distance allows for greater temporal dispersion of postsynaptic events so they can be identified as arising from different synapses. Preservation of the postsynaptic nerves is required for extracellular recording of compound postsynaptic responses with suction electrodes. The compound postganglionic responses enable one to monitor the efficacy of graded presynaptic stimulation [c.f. Figure 4 in Springer et al. (2015)]. In earlier work, some recordings were made at room temperature in order to slow axonal conduction velocities and assess subsets of preganglionic axons (Li and Horn, 2006). The limitation of this approach is that it also perturbs synaptic transmission. For this reason, the present experiments were all done at 36°C to approximate normal body temperature. Another important aspect of preparing ganglia for patch recording is the dissection of connective tissue. For microelectrode recordings, one splits the connective tissue sheath surrounding the ganglion and tightly pins it out, like the head of a drum. This is essential for piercing remaining barriers with a sharp electrode. Patch recording requires an entirely different approach. After isolating the ganglion, the connective tissue is carefully nicked with fine scissors and then entirely stripped away, like removing a glove. The isolated ganglion is then placed in a recording chamber and fitted with suction electrodes (Figure 1B). At this stage, the ganglion is treated with collagenase, allowed to rest, and then used for recording. Installing the tight-fitting suction electrodes before adding the collagenase is essential because it protects the integrity of the nerves, which become very delicate after enzyme digestion. The ganglion is mechanically stabilized for recording by the suction electrodes and by anchoring it with one or two stainless minutien pins (Fine Science Tools, Foster City, CA, United States) (Figure 1B). The pins are cut to shorten them, bent into L- or J-like hooks and placed gently beside and over the main body of the ganglion prior to enzyme treatment.

### Recording techniques and solutions

Ringer solution for dissection and recording contained (in mM): 146 NaCl, 4.7 KCl, 20 HEPES, 0.6 MgSO_4_, 1.6 NaHCO_3_, 0.13 NaH_2_PO_4_, 2.5 CaCl_2_, 7.8 glucose, pH adjusted to 7.4 with NaOH, osmolality adjusted to 314 mOsm with dH_2_O and bubbled with 100% O_2_. For enzyme digestion Ringer was replaced by a solution consisting of Leibovitz’s L15 medium (Gibco/Fisher Scientific, Pittsburgh, PA, United States) supplemented with 14 mM NaHCO_3_ and 10 mg/ml collagenase Type 3 (Worthington, Lakewood, NJ, United States). The recording chamber and ganglion with enzyme solution was then placed uncovered for 60–90 min into a humidified tissue culture incubator (37°C, 5% CO_2_). After mounting the recording chamber on a fixed stage upright microscope (Zeiss Axioskop), the enzyme solution was washed away with oxygenated Ringer and the preparation was allowed to rest for 30 min before recording.

Patch electrodes were fabricated from thick-walled borosilicate capillaries (1.5 mm OD, 0.86 mm ID, #1B150F-4, World Precision Instruments (WPI), Sarasota, FL, United States) on a P-87 puller (Sutter Instrument, Novato, CA, United States). Pipettes resistances were 2.8–3.3 MΩ when filled with internal solution (in mM: 94 K^+^-gluconate, 30 KCl, 10 HEPES, 0.2 EGTA, 10 phosphocreatine di tris salt, 4 Mg_2_ATP, 0.3 Na_2_GTP, pH adjusted to 7.3 with KOH and osmolality adjusted to 300 mOsm with sucrose). Data was corrected after experiments to account for a calculated tip potential of + 14 mV.

Extracellular stimulation and recording with suction electrodes fabricated from polyethylene catheter tubing was as previously described (Li and Horn, 2006). Presynaptic stimuli consisting of 30–100 μs unipolar rectangular current pulses were delivered through an isolator (A360; WPI) gated by a pulse train generator (A300; WPI). Differential recordings of extracellular compound action potentials were made with Grass AC preamplifiers (Model P55, Astro-Med, West Warwick, RI, United States) with gain set to 1,000 or 10,000 and bandpass filters at 0.1 Hz and 3 kHz.

Patch recordings were made under visual guidance (Figure 1C) through a 40× water immersion objective (Zeiss Achroplan 40×/0.75 W #44 00 90) and TillVision imaging system with IMAGO charge-coupled device (CCD) camera. Signals were fed through a CV-7B head stage to a MultiClamp 700B amplifier (Molecular Devices, San Jose, CA, United States). The electrode and head stage assembly was mounted on a Luigs & Neumann Mini 25 manipulator (Ratingen, Germany). Resistances were 1–3 giga-Ohms during seal formation. In cell-attached and whole-cell voltage-clamp recordings the holding potential was set to −51 mV. In whole-cell voltage clamp mode, series resistance (R_series_) was monitored throughout the recording and R_series_ compensation was set to 85%. Recordings in which R_series_ exceeded 25 meg-Ohms were excluded from analysis. Experimental control and data logging used a Digidata 1440A interface together with pClamp 10 and MultiClamp Commander software (Molecular Devices). While monitoring R_series_ during voltage clamp recordings, measurements of membrane capacitance (C_m_) and input resistance (R_input_) were obtained using the cell membrane monitoring routine built into pClamp.

### Data analysis

Data analysis utilized pClamp 10, Igor Pro 7 (WaveMetrics, Lake Oswego, OR, United States) and Prism 9 (GraphPad Software, La Jolla, CA, United States). Amplitudes of synaptic currents were measured from the baseline to the peak. In cases of temporal overlap between excitatory postsynaptic currents (EPSCs) with different latencies, baselines were extrapolated by fitting an exponential function to the decay of the first EPSC. Peak synaptic conductance (g_syn_) was calculated from synaptic currents using Ohm’s law with a corrected membrane holding potential of −65 mV and a reversal potential of −11 mV. When calculating means and standard deviations of EPSCs, the rare trials that failed to elicit a response were excluded. Normality tests and Spearman correlation calculations were done with Prism 9. Grouped data are expressed as the mean ± standard deviation except where noted.

## Results

This paper presents data from 20 neurons from 20 rats where straddling synapses were initially detected in extracellular on-cell recordings and then characterized further with intracellular whole cell recordings in the voltage-clamp and current-clamp modes. Patch recording in these configurations has not to our knowledge been used before to study the physiology of converging synapses in paravertebral sympathetic neurons. These experiments were part of a larger series, unpublished at present, whose goal was to assess with intracellular recording the properties of individual nicotinic synapses on sympathetic SCG neurons. In all experiments, temperature was held at 36°C and the presynaptic nerve was stimulated repetitively at 1 Hz. The slow rate of stimulation was chosen to minimize possible confounding effects introduced by facilitation, depression, and non-nicotinic synaptic mechanisms. As in previous microelectrode studies of paravertebral sympathetic ganglia, individual converging synaptic inputs were distinguished by their distinct presynaptic stimulus thresholds and their latencies (Dodd and Horn, 1983; Hirst and McLachlan, 1984; Karila and Horn, 2000; McLachlan, 2003; Rimmer and Horn, 2010; Springer et al., 2015). The modest extent of convergence in sympathetic ganglia makes this approach feasible. It has been estimated that an average of 8.7 nicotinic synapses converge on rat SCG neurons, based on sharp microelectrode recordings of membrane potential together with ventral root stimulation (Purves and Lichtman, 1985).

**FIGURE 2:**
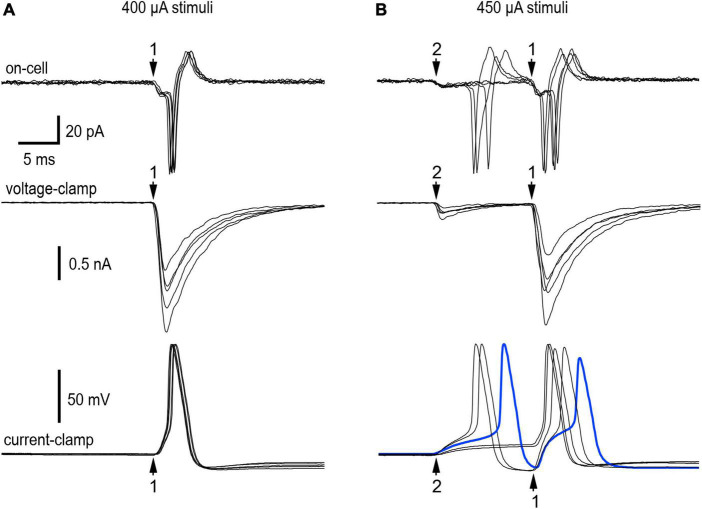
Resolution of convergence between a primary synapse and a straddling synapse. Extracellular on-cell recordings (top panels) and intracellular whole-cell recordings under voltage-clamp (middle panels) and current-clamp (bottom panels) made while stimulating the preganglionic cervical sympathetic trunk (CST) at two different shock intensities. **(A)** 400 μA shocks selectively recruited synapse 1–a strong primary synapse that never failed during repetitive stimulation at 1 Hz. **(B)** Raising the stimulus strength to 450 μA recruited synapse 2, a much weaker synapse that straddled threshold during repeated trials at 1 Hz. At 450 μA, single shocks could elicit double spikes resulting from convergence. Each panel contains five traces chosen to reflect variability in responses. See text for additional details about all the responses. In the on-cell recordings (top panels) one sees evidence of excitatory postsynaptic potentials (EPSPs) and the action potentials they trigger. The voltage-clamp recordings of excitatory postsynaptic currents (EPSCs) (middle panels) show the temporal correspondence with extracellular recordings and the markedly different EPSC amplitudes at synapses 1 and 2. Under current-clamp (bottom panels) there was little variation in the onset of action potentials driven by synapse 1. This contrasted with the smaller EPSPs at synapse 2, where the onset of action potentials was more variable. Note the heavy blue trace showing an action potential evoked by synapse 2 very shortly before the activation of synapse 1. This resulted in the second action potential having a slower upstroke and lower amplitude. Experiment 11/9/18 cell 2.

The extracellular cell-attached recordings in Figure 2 illustrate responses from 2 nicotinic synapses with distinct presynaptic stimulus thresholds and latencies (experiment 11/9/18 cell 2). In cell-attached recordings, the currents recorded at the patch reflect unclamped intracellular EPSPs and action potentials. We therefore refer to these extracellular current recordings as on-cell or extracellular EPSPs and action potentials. Five sets of 30 stimuli at 1 Hz were used to find the minimal stimulus intensity needed to recruit synapse 1 and then synapse 2. In response to 400 μA stimuli, 30 of 30 shocks elicited suprathreshold on-cell EPSPs at synapse 1 (Figure 2A, top). Finding that all EPSPs at synapse 1 were suprathreshold in strength conforms to the definition of a primary nicotinic synapse (Karila and Horn, 2000). Each postsynaptic response began with a small downward extracellular EPSP that led to a much larger current associated with an action potential. The latency from the stimulus to onset of the extracellular EPSP was 29.5 ms. When the stimulus was reduced slightly, only 36 of 60 stimuli evoked suprathreshold EPSPs and the remaining 24 stimuli failed to evoke any response. We interpret this as the intermittent failure of axonal stimulation to initiate a presynaptic action potential. These observations indicate that 400 μA was the minimum stimulus required for reliable activation of synapse 1. Raising the stimulus to 450 μA recruited synapse 2, whose latency was 17.0 ms. Under these conditions, 29 of 30 stimuli evoked extracellular EPSPs at synapse 2 with 1 failure. Fourteen of the 29 EPSPs (48%) triggered action potentials and the other 15 EPSPs (52%) were subthreshold in strength. Because the latency of synapse 2 was less than that of synapse 1, the properties of synapse 2 were not influenced by synapse 1. The extracellular responses elicited by selective activation of synapse 2 indicate that the ability of a synapse to straddle threshold is not an artifact of intracellular recording. The data from this neuron also shows that a single shock can elicit double spiking by activating 2 synapses. Increasing the stimulus above 450 μA recruited a third synapse that was not included in the analysis.

After going from the on-cell to the whole-cell recording configuration, we assessed EPSCs and EPSPs produced by synapses 1 and 2 (Figure 2, middle and bottom traces). Under voltage-clamp the mean EPSC amplitudes were −1272 ± 260 pA (*n* = 95) at synapse 1 and −232 ± 123 pA (*n* = 55) at synapse 2. Under current-clamp EPSPs evoked by minimal stimulation (400 μA) to selectively activate synapse 1 triggered action potentials in 30 of 30 trials, consistent with the extracellular observations. Raising the stimulus strength to 450 μA evoked suprathreshold EPSPs at synapse 2 in 20 of 30 trials (67%) and subthreshold EPSPS in 10 trials (33%), with no failures of stimulation. This provides intracellular confirmation that a single shock can evoke two action potentials by activating two converging synapses. When activated in isolation synapse 1 never failed to evoke an action potential, and the spikes were nearly superimposable (Figure 2A). This contrasts with synapse 2, where EPSPs straddled threshold and the timing of action potential initiation was quite variable (Figure 2B). As a result of this variable timing, action potentials triggered by synapse 2 sometimes occurred within 2–5 ms of the EPSP at synapse 2 (see heavy blue trace in Figure 2B, bottom). In 6 of 11 trials where this happened, the spike at synapse 2 occluded generation of the second spike at synapse 1. This is not surprising given that action potentials undergo a refractory period caused by Na^+^ channel inactivation. Signs of inactivation can be seen in Figure 2B (blue trace) where the spike at synapse 1 is reduced in amplitude and upstroke velocity. Nonetheless the difference in synaptic strength at the two synapses was very large. EPSCs were 5.5 times larger and 8.4 standard deviations greater at synapse 1 than 2.

Taking the intracellular and extracellular data into account, how then would one categorize synapse 1 and 2 in terms of the primary-secondary nomenclature that originally defined synapses as being either subthreshold or suprathreshold in strength (Karila and Horn, 2000)? Synapse 2 does not fit into either of the original categories because it straddles threshold when stimulated in isolation. This is distinct from synapse 1 which was much stronger than synapse 2 and always suprathreshold when stimulated in isolation. These criteria indicate synapse 1 should be classified as a primary synapse and synapse 2 as a straddling synapse.

**FIGURE 3:**
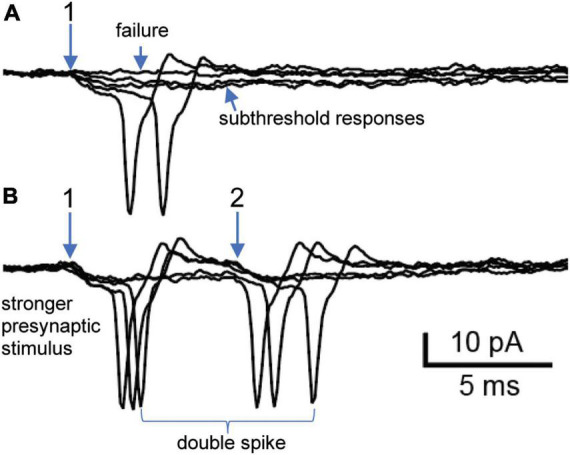
Extracellular recording of double spiking driven by two straddling synapses. **(A)** Selective activation of synapse 1. Five responses at synapse 1 include 2 subthreshold responses, 2 suprathreshold responses and 1 stimulus failure. Traces taken from a larger trial at 1 Hz (see text for description of all responses). **(B)** Raising the presynaptic stimulus strength recruited synapse 2 and eliminated failures at synapse 1. Note that synapse 2 also straddled threshold by generating subthreshold and suprathreshold responses. Experiment 6/2/16 cell 4.

**FIGURE 4:**
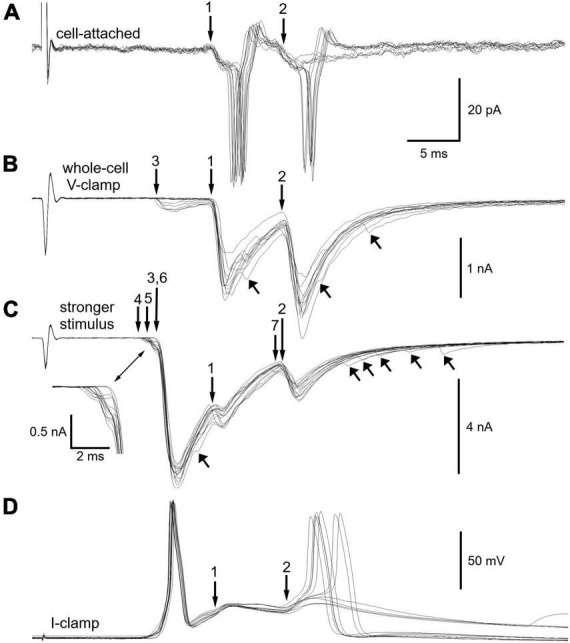
Fractionation of 7 synapses that converge on a sympathetic neuron. **(A)** Additional extracellular on-cell recordings from the same neuron illustrated in Figure 3. **(B)** Intracellular voltage-clamp recordings of synaptic currents after breaking into the cell. Presynaptic stimulus strength unchanged from panel **(A)**. The records show families of excitatory postsynaptic currents (EPSCs) whose latencies match those of responses 1 and 2 in panel **(A)**. The smaller EPSCs at synapse 3 were not detected in the on-cell recording. Upward arrows point to examples of asynchronous EPSCs present in some trials. **(C)** After increasing the presynaptic stimulus strength, a total of 7 synaptic components were resolved based on the latencies and magnitudes of EPSCs. Inset shows the small EPSCs at the foot of the large EPSCs associated with synapse 6. Upward arrows point to additional asynchronous EPSCs. **(D)** Switching the amplifier to current-clamp mode reveals EPSPs that drive pairs of action potentials. Note that the larger synaptic events (6, 1, 2) obscure the smaller ones. Note also that the double spiking differs from that in panel **(A)**. Synapses 6 and 2 rather than synapses 1 and 2 now trigger action potentials. See text for additional detail.

Next, we consider a neuron where 7 converging nicotinic synapses were resolved by their distinct presynaptic stimulus thresholds and latencies (experiment 6/2/16 cell 4). This cell provides another example where a single shock could lead to double spiking driven by converging nicotinic synapses. Initial extracellular on-cell recording from this neuron revealed 2 synapses that both straddled threshold (Figure 3). Synapse 1 had a lower presynaptic stimulus threshold and a latency of 16.5 ms. The traces in Figure 3A illustrate extracellular examples of stimulus failures, subthreshold EPSPs, and suprathreshold EPSPs. In 37 trials, there were 9 failures. In the 28 trials that evoked EPSPs, 20 (71%) were suprathreshold while 8 (29%) were subthreshold. Increasing the stimulus strength recruited synapse 2 (Figure 3B), which had a latency of 23 ms. With the stronger stimuli used to recruit synapse 2 the failures at synapse 1 were eliminated, thereby showing that they arose from failures of stimulation and not failures of transmitter release. In 19 trials, 11 responses (57%) at synapse 2 were suprathreshold in strength and 8 (43%) were subthreshold. Although both synapses straddled threshold, only synapse 1 could be observed in isolation (Figure 3A). One might therefore question whether the efficacy of synapse 2 was altered by its temporal interaction with synapse 1. Resolving this question required intracellular measurements (Figure 4).

After breaking into the cell under voltage-clamp (Figure 4B) while maintaining the same presynaptic stimulus strength, a total of 3 synapses became evident. The latencies of extracellular EPSPs at synapses 1 and 2 (Figure 4A) correspond to the latencies of the intracellular EPSCs labeled 1 and 2 (Figure 4B). Peak synaptic current amplitudes were −1391 ± 236 pA at synapse 1 and −1520 ± 252 pA at synapse 2. Given that the mean currents for each synapse are within 1 standard deviation of each other and that synapse 1 straddles threshold when stimulated in isolation (Figure 3A), it seems highly likely that synapse 2 would also straddle threshold if it could be stimulated in isolation.

After increasing the presynaptic stimulus strength (Figure 4C) a total of 7 synaptic components were identified. The family of EPSCs at each synapse had a distinct latency and distinct average amplitude (Table 1). Given complexity of waveforms arising from seven converging synapses, one might ask whether trial to trial fluctuations in the latencies of responses obscure additional synaptic inputs. Although impossible to rule out this concern with complete certainty, this problem seems unlikely to confound the results because fluctuations in latency were small. As seen in Figures 4A–C, fluctuations in the latency of individual synaptic components were less than 0.5 ms, while the conduction delays of individual synaptic components ranged from 11 to 23 ms. Due to the length of presynaptic CST (Figure 1B) and the slow conduction velocities (<1 m/s) of preganglionic axons (Li and Horn, 2006), which are unmyelinated C fibers, the jitter in postsynaptic responses accounts for less than 5% of the total synaptic latency.

**TABLE 1:** Strength classification of synapses converging on one cell.

Synapse number	Peak EPSC (pA) mean ± SD (*n*)	Peak g_syn_ (nS)	Synapse strength% threshold-g_syn_	Strength classification
1	−1391 ± 236 (37)	25.8	100	Straddling
2	−1520 ± 252 (13)	28.2	109	Straddling
3	−156 ± 57 (14)	2.9	11	Secondary
4	−148 ± 36 (7)	2.7	10	Secondary
5	−381 ± 99 (6)	7.0	27	Secondary
6	−5355 ± 448 (20)	99.2	384	Primary
7	Not resolved	–	–	Secondary

Data in this table are from the neuron illustrated in Figure 4. All currents were measured at a corrected holding potential of −65 mV. Synapses 1 and 2 correspond to synapses 13 and 14 in Table 2. Excitatory postsynaptic currents (EPSCs) at synapse 7 were too small to permit a reliable estimate of their amplitude.

The strength of synapse 6 in Figure 4C is notable because it produced EPSCs that were >3.5 times larger (>15 standard deviations) than those at synapses 1 and 2, which straddled threshold. Thus, the safety factor for suprathreshold transmission at synapse 6 is very high, consistent with the example shown in Figure 2 and with the original definition of a primary synapse (Karila and Horn, 2000). Asynchronous EPSCs having low amplitudes were also evident in this neuron (Figures 4B,C).

At this point we examined the membrane potential response to strong stimuli that activated all 7 synapses by switching from whole cell voltage-clamp to the current-clamp mode (Figure 4D). In these recordings evidence of the weaker synapses is obscured by the larger responses at synapses 1, 2, and 6. Interestingly, the cell double-spiked, just as in the initial extracellular recording. However, now the paired spiking was driven by synapses 6 and 2 rather than by synapses 1 and 2. This shows that the action potentials evoked by suprathreshold EPSPs at synapse 6 had the effect of completely occluding action potentials at synapse 1. The occlusion likely results from reduced excitability due to Na^+^ channel inactivation during the refractory period and from increased K^+^ conductance during the spike afterpotential (McAfee and Yarowsky, 1979; Galvan and Sedlmeir, 1984; Wheeler et al., 2004).

Table 1 summarizes the average magnitude and variability of each synaptic input to the cell illustrated in Figure 4 in terms of its underlying peak EPSC amplitude, standard deviation, and the associated synaptic conductance (g_syn_). Given that the extracellular responses evoked at synapses 1 and 2 straddle threshold, the associated synaptic currents provide a useful index of synaptic strength. Converting the mean peak current to a nicotinic conductance is convenient because it is independent of the holding membrane potential where currents are measured and is often used as an input parameter for computational models and dynamic clamp experiments. Threshold synaptic conductance (threshold-g_syn_) defines synaptic strength as the minimum nicotinic synaptic conductance required to reach firing threshold for an action potential (Schobesberger et al., 2000; Kullmann et al., 2004; Wheeler et al., 2004). Earlier work estimated threshold-g_syn_ using computational models and a virtual nicotinic synapse whose strength was systematically varied to find threshold. The straddling synapses described here enable one to estimate threshold-g_syn_ independent of simulations and dynamic clamp methods. By using the average synaptic conductance of a straddling synapse as an estimate of threshold-g_syn_ one can then calculate the relative strengths of the other synapses (Table 1) and then classify them as primary, straddling, or secondary. This approach indicates that synapse 6 was a primary synapse whose strength was nearly four times threshold-g_syn_, that synapses 1 and 2 were straddling synapses and that synapses 3, 4, 5, and 7 were secondary synapses whose strengths were <30% threshold-g_syn_. Unlike straddling EPSPs, secondary EPSPs never trigger action potentials unless they summate with other EPSPs.

The average g_syn_ of 22 straddling synapses was 9.8 ± 7.5 nS (mean ± SD). Table 2 summarizes the properties of these straddling synapses and the 20 neurons from 20 animals where they were studied. They were identified as straddling through extracellular on-cell recording and then assessed by intracellular voltage-clamp and current-clamp measurements. Synapse 2 in Figure 2 corresponds to cell 5 in Table 2. Synapses 1 and 2 in Figures 3, 4 and in Table 1 correspond to synapses 13 and 14 in Table 2. Although we did not set out to study differences between sexes and strains, the data demonstrate that straddling synapses were evident in 3 female CD rats, 13 male CD rats, and 3 male SHR rats.

**TABLE 2:** Summary of straddling synapses.

Synapse number	Date	Cell	Sex	Strain	Age (days)	On-cell straddling [% suprathreshold (in *n* trials)]	V_rest_ (mV)	R_input_ (meg-Ω)	R_series_ (meg-Ω)	C_m_ (pF)	I_syn_ (pA) mean ± SD (*n*)	g_syn_ (nS) mean
1	1/25/2018	c2	M	CD	87	83.3 (6)	−61	258	6.2	127	−779 ± 180 (13)	14.4
2	4/13/2018	c1	M	CD	60	83.3 (6)	−56	234	7.1	74	−564 ± 158 (26)	10.4
3	4/30/2018	c5	M	CD	63	73.3 (30)	−56	577	7.9	61	−252 ± 36 (5)	4.7
4		c5				87.5 (8)					−253 ± 90 (29)	4.7
5	11/9/2018	c2	M	CD	109	37.9 (29)	−57	662	6.9	51	−232 ± 123 (55)	4.3
6	1/11/2017	c2	F	CD	65	9.5 (42)	−57	857	5.3	69	−295 ± 126 (34)	5.5
7	1/13/2017	c3	F	CD	67	95.2 (21)	−68	551	4.6	81	−694 ± 190 (37)	12.9
8	1/27/2017	c2	F	CD	63	41.9 (31)	−51	602	4.9	61	−215 ± 84 (52)	4.0
9	12/21/2017	c2	M	SHR	73	5.3 (57)	−59	703	9.0	65	−328 ± 125 (48)	6.1
10	1/28/2016	c2	M	CD	66	64.3 (28)	−60	312	12.4	95	−1339 ± 295 (65)	24.8
11	2/19/2016	c1	M	SHR	67	74.1 (54)	−65	346	6.5	77	−493 ± 119 (120)	9.1
12	2/19/2016	c5	M	SHR	67	33.3 (12)	−52	721	7.4	34	−196 ± 90 (80)	3.6
13	6/2/2016	c4	M	CD	66	80.4 (51)	−78	393	6.5	80	−1391 ± 236 (37)	25.8
14		c4				57.9 (19)					−1520 ± 252 (13)	28.2
15	6/6/2016	c5	M	SHR	161	20.0 (10)	−58	664	4.9	76	−182 ± 100 (17)	3.4
16	6/8/2016	c1	M	CD	65	62.5 (32)	−59	645	9.9	51	−244 ± 100 (47)	4.5
17	8/24/2016	c1	M	CD	65	10.7 (28)	−62	498	6.6	67	−231 ± 107 (29)	4.3
18	9/13/2016	c4	M	CD	64	59.4 (32)	−62	390	4.9	79	−593 ± 193 (61)	11.0
19	9/16/2016	c2	M	CD	67	69.2 (13)	−59	703	9.5	60	−398 ± 144 (33)	7.4
20	9/30/2016	c4	M	CD	69	90.0 (30)	−58	1200	4.6	46	−205 ± 35 (23)	3.8
21	10/13/2016	c3	M	CD	73	67.9 (53)	−57	263	7.4	77	−739 ± 208 (90)	13.7
22	7/28/2015	c1	M	CD	49	62.1 (29)	−68	293	10.2	88	−449 ± 127 (70)	8.3
				Mean	73.3	57.7	−60.2	544	7.1	71.0	−527	9.8
				SD	23.7	27.5	6.1	242	2.1	19.9	407	7.6

In light of the trial-to-trial fluctuations in response amplitudes one might expect that the mean g_syn_ of a straddling synapse could underestimate or overestimate threshold-g_syn_ and that this would be reflected in the synaptic efficacy in triggering action potentials. For example, the mean g_syn_ of a synapse that triggers action potentials in 90% of trials would overestimate threshold-g_syn_. Conversely, the mean g_syn_ of a synapse that triggers action potentials in 10% of trials would underestimate threshold-g_syn_. To assess this possibility, we counted subthreshold and suprathreshold responses in extracellular and intracellular recordings. In extracellular on-cell recordings, 57.7 ± 27.5% of trials elicited spikes (Table 2, *n* = 22 synapses, range 9.5–95.2%). In intracellular current-clamp recordings, 61.0 ± 27.0% of trials elicited spikes (*n* = 17 synapses, range 16.7–96.6%). These data indicate that the overall average g_syn_ of 9.8 nS (Table 2) is a slight overestimate of threshold-g_syn_ that ideally would elicit spikes in 50% of trials.

**FIGURE 5:**
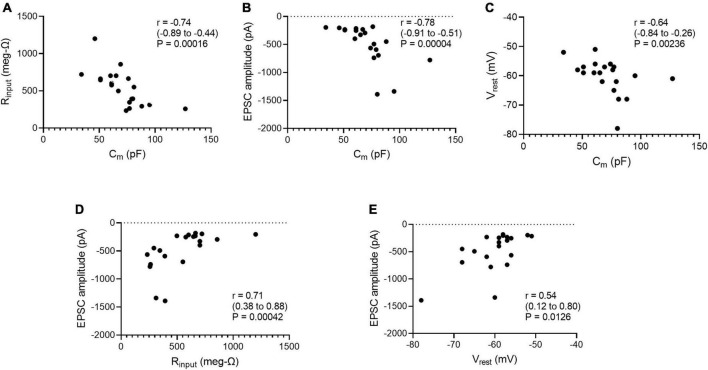
Whole cell capacitance correlates with **(A)** input resistance, **(B)** peak excitatory postsynaptic current (EPSC) amplitudes at straddling synapses, and **(C)** resting membrane potential. Peak EPSC amplitudes at straddling synapses correlate with **(D)** input resistance and **(E)** resting membrane potential. C_m_ was taken as a measure of cell size. Spearman correlation coefficients (r) were calculated for each scatter plot along with 95% confidence intervals and 2-tailed *P*-values. C_m_, R_input_, and EPSCs were measured under voltage-clamp. V_rest_ was measured under-current clamp. All data are from Table 2.

Age and cell size are factors that could account for some of the cell-to-cell variability in the g_syn_ of straddling synapses, input resistance (R_input_) and resting potential (V_rest_). For example, cells might grow over time and larger cells would have lower R_input_ and thus require stronger EPSPs to reach threshold. One might also expect that cells with lower resting potentials would require larger synaptic currents to reach threshold. To examine these possibilities, we examined the data distributions of C_m_, R_input_, V_rest_, and peak EPSC amplitude, and then looked for correlations between them. The D’Agostino-Pearson normality test indicated that R_input_ measurements were compatible with the null hypothesis (H_0_) of a normal distribution, but that measurements of C_m_, EPSC amplitude and V_rest_ were incompatible with this H_0_. We therefore calculated correlation coefficients (r) using the non-parametric Spearman method, together with 2-tailed *P*-values and 95% confidence intervals for evaluating the null hypothesis that *r* = 0. Taking whole cell capacitance (C_m_) as an index of cell size, the data were incompatible with correlations between age and either C_m_ (*r* = −0.071, *P* = 0.765), EPSC amplitude (*r* = 0.086, *P* = 0.717), R_input_ (*r* = 0.275, *P* = 0.239), or V_rest_ (*r* = 0.007, *P* = 0.974). By contrast, there were negative correlations between C_m_ and R_input_ (Figure 5A), C_m_ and EPSC amplitude (Figure 5B), and C_m_ and V_rest_ (Figure 5C). These results are consistent with the expectation that larger cells would have more open channels at rest and therefore a lower R_input_. They also conform to the idea that larger synaptic currents would be required to depolarize cells with lower R_input_ to reach threshold. The correlation between C_m_ and V_rest_ under current clamp may reflect the possibility that smaller neurons are more susceptible to recording damage. In addition, positive correlations incompatible with H_0_ were also seen between R_input_ and EPSC amplitude (Figure 5D) and between V_rest_ and EPSC amplitude (Figure 5E). This supports the idea that cells with higher R_input_ or lower V_rest_ require larger EPSCs to reach threshold.

## Discussion

Several years ago, high resolution patch recording of synaptic transmission became possible in the isolated intact SCG (Springer et al., 2015). Using this approach (Figure 1), we set out to explore the properties of individual synapses that converge on individual sympathetic neurons. Along the way, it became evident that on-cell recordings established before breaking into the whole-cell configuration revealed important information about the strength of synapses.

The detection in on-cell extracellular recordings of nicotinic synapses whose strength straddles threshold during repetitive 1 Hz stimulation demonstrates that such straddling is not an artifact of intracellular recording (Figures 2–4 and Table 2). In addition, the present results replicate double spike responses to single presynaptic stimuli seen previously in microelectrode recordings (Rimmer and Horn, 2010). In the earlier work it was unclear whether double spiking reflected converging synapses or repetitive firing. The present results show that double spiking arises from converging synapses with different latencies (Figures 2B, 3B, 4A,D) and not to repetitive firing during sustained depolarization or from intracellular recording damage (Figures 2B, 3B, 4A). In current-clamp recordings, repetitive firing was never seen in response to EPSPs evoked at one synapse, regardless of their strength.

At synapses where nicotinic EPSPs straddle threshold, the underlying synaptic currents provide an estimate of threshold-g_syn_. Using this approach, threshold-g_syn_ in the intact SCG was 9.8 ± 1.6 nS (mean ± standard error, range 3.4–28.2 nS, *n* = 22). This closely resembles previous estimates based on a computational model and on dynamic clamp experiments from dissociated sympathetic neurons. In a conductance-based model tuned to replicate properties of bullfrog sympathetic B neurons, threshold-g_syn_ = 10.1 nS (Schobesberger et al., 2000). When dynamic clamp was used to produce virtual nicotinic EPSPs in recordings from dissociated bullfrog sympathetic neurons, threshold-g_syn_ = 14.7 ± 1.7 nS (mean ± standard error) (Wheeler et al., 2004). Other dynamic clamp experiments with dissociated bullfrog sympathetic neurons revealed phenotypic differences in threshold-g_syn_ measurements from identified secretomotor B neurons [17.1 ± 1.2 nS, (mean ± standard error)] and vasomotor C neurons (3.3 ± 0.3 nS, mean ± standard error) (Kullmann and Horn, 2010b). This is interesting because it suggests that phenotypic specialization may explain some of the variability seen in our analysis of straddling synapses in the rat SCG. In dynamic clamp experiments using dissociated rat SCG neurons threshold-g_syn_ = 7.0 ± 0.9 nS (mean ± standard error) (Kullmann et al., 2016). Other experiments on rat SCG neurons suggest that cell specific differences in repetitive firing dynamics may also be associated with variability seen in threshold-g_syn_ (Springer et al., 2015).

Normalizing synaptic conductance measurements to threshold-g_syn_ enables one to appreciate the range of synaptic strength expressed by converging synapses and between different neurons. Whether expressed in terms of size as synaptic current or synaptic conductance, or in terms of strength as %-threshold-g_syn_, the 6 converging synapses in Table 1 produced responses whose magnitude spanned a 38-fold range. In moving beyond the original primary-secondary synapse dichotomy (Karila and Horn, 2000), straddling synapses must now be considered. This conclusion supports an earlier idea by Bratton et al. (2010). They devised an ingenious method to estimate the strength of EPSPs recorded *in vivo* from lumbar chain ganglia in the rat with intracellular current-clamp methods. By injecting hyperpolarizing currents, they estimated the extent to which suprathreshold EPSPs could be disrupted. Although indirect, this approach led them to conclude that the synapses that drive firing in different neurons varied in their strength. They also proposed that the distribution of synaptic strength was continuous rather than lumped into two discrete groups. Although this concept is consistent with our data, more work will be required to fully delineate the strength distribution of converging synapses.

Our interest in primary and secondary nicotinic synapses began with an analysis of convergence in bullfrog secretomotor neurons using microelectrode recordings (Karila and Horn, 2000). Given the tools of the time, it was simplest to ask whether a synapse was very strong with a high safety factor and thus a primary synapse that never failed to elicit an action potential–or whether it was weaker and thus secondary. Now with the advantage of hindsight one can see that straddling synapses also exist in bullfrog sympathetic ganglia. Figure 1 in Karila and Horn (2000) illustrates a bullfrog B-type neuron with 2 converging synapses. The primary synapse invariably evokes an action potential and is strong enough to deform the spike afterhyperpolarization. The weaker synapse is generally subthreshold in strength and does not deform the spike afterhyperpolarization, indicating its underlying synaptic conductance is much smaller than that of the primary synapse. However, fluctuations in EPSPs at the weaker synapse enable it to trigger spikes in some trials! We would therefore now label it as a straddling synapse and not a secondary synapse. Weaker secondary synapses are evident in other data shown in the Karila and Horn’s (2000) paper.

Understanding the strength of converging nicotinic synapses in sympathetic ganglia has important implications for ganglionic integration. Postulating that convergence follows an *n* + 1 rule and that summation of secondary EPSPs can drive firing enabled the construction of computational models (Karila and Horn, 2000; Wheeler et al., 2004; Kullmann and Horn, 2006, 2010a; Kullmann et al., 2016). The models predicted that paravertebral sympathetic ganglia function as use-dependent amplifiers of preganglionic activity. Simply stated, more action potentials leave the ganglion than enter it, because of synaptic amplification. Ganglionic gain could be important in normal cardiovascular physiology and in hypertension (Guyenet, 2006; Fadel, 2008; Joyner et al., 2008; Esler, 2010, 2014; Malpas, 2010). In baroreceptor coupled sympathetic circuits that control blood pressure, functional gain is important. Variable synaptic gain in ganglia could play a role. Hypertension in many individuals is associated with sympathetic hyperactivity. What, if any, role does a remodeling of ganglionic gain play in generating sympathetic hyperactivity?

In closing, the identification of straddling synapses demonstrates that previous models of ganglionic integration are no longer sufficient. However, this does not disprove the synaptic gain hypothesis or show that ganglia behave as simple relays rather than as integrative centers. It means that more nuanced models incorporating synapses of different strengths will be required to elucidate ganglionic computation and its contribution to normal physiology and pathophysiology.

## Data availability statement

The original contributions presented in this study are included in the article/supplementary material, further inquiries can be directed to the corresponding author.

## Ethics statement

The animal study was reviewed and approved by IACUC, University of Pittsburgh.

## Author contributions

PK was primarily responsible for conducting the experiments. Both authors contributed to the design of experiments, analysis, and writing the manuscript.
